# Quality of life in university professors: association with components of spirituality and professional achievement

**DOI:** 10.3389/fpsyg.2025.1735377

**Published:** 2026-01-05

**Authors:** Cezimar Correia Borges, Renata Custódio Maciel, Uitarany do Prado Lemes, Matias Noll, Maria Alves Barbosa, Celmo Celeno Porto, Patrícia Roberta dos Santos, Giancarlo Lucchetti, Weder Alves Silva, Celaine Ribeiro, Vicente Aprigliano, Marina Silveira, Gerson Ferrari, Alberto Souza Sá Filho

**Affiliations:** 1 Institute of Biological Sciences - Goiás State University, Itumbiara, Brazil; 2 Unicerrado - University Center Goiatuba, Goiatuba, Brazil; 3 Goiano Federal Institute - Ceres Campus, Ceres, Brazil; 4 Program in Health Science – Federal University of Goiás, Goiânia, Brazil; 5 Zarns School of Medicine – Itumbiara Goiás, Itumbiara, Brazil; 6 School of Medicine – Federal University of Juiz de Fora, Juiz de Fora, Brazil; 7 Graduate Program in Human Movement and Rehabilitation (PPGMHR) – Evangelical University of Goias – UniEVANGÉLICA, Goiânia, Brazil; 8 Escuela de Ingeniería de Construcción y Transporte, Pontificia Universidad Católica de Valparaíso, Avda Brasil, Valparaíso, Chile; 9 Núcleo de Investigación en Ciencias del Movimiento, Universidad Arturo Prat, Iquique, Chile; 10 Facultad de Ciencias de la Salud, Universidad Autónoma de Chile, Providencia, Chile; 11 Universidad de Santiago de Chile (USACH), Escuela de Ciencias de la Actividad Física, el Deporte y la Salud, Santiago, Chile

**Keywords:** college professor, job satisfaction, psychological framework, quality of life, spirituality

## Abstract

**Background:**

University professors face considerable workload pressures and professional demands that can negatively affect their quality of life (QoL). Positive emotions associated with spirituality and religiousness (S/R) have been linked to enhanced QoL in various populations; however, limited research has explored this relationship among university professors.

**Objective:**

To examine the association between spirituality/religiousness and quality of life among university professors, identifying the most influential spiritual facets and relevant sociodemographic predictors.

**Methods:**

This cross-sectional study included 213 professors from a public university in Goiás, Brazil. Participants completed the WHOQOL-BREF (QoL) and WHOQOL-SRPB (S/R) instruments, both validated for the Brazilian population.

**Results:**

Seven of the eight S/R facets showed significant positive correlations (*p* ≤ 0.01) with QoL domains. The strongest associations were observed in the psychological domain, particularly for hope and optimism (*r* = 0.67), spiritual strength (*r* = 0.62), and faith (*r* = 0.59). Regression analyses indicated that these facets were the strongest independent predictors of overall QoL (*R*^2^ = 0.28). Professional and sociodemographic factors such as academic degree, job satisfaction, professional recognition, and income were also associated with higher QoL, while holding multiple employment contracts was inversely related to it.

**Conclusion:**

Higher levels of S/R were positively associated with QoL among university professors. These findings highlight the potential value of integrating spirituality-based activities into university wellness programs as effective strategies to promote well-being and help faculty manage occupational demands.

## 1 Introduction

In many populations, daily life is shaped by intense socioeconomic and cultural pressures. Among university professors, these pressures are compounded by multiple academic responsibilities, teaching, research, service, and administrative duties, which can become a constant challenge and, at times, compromise their health and quality of life (QoL) (Dias et al., 2018; Koetz et al., 2013; de Oliveira et al., 2012). Previous studies investigating QoL among university faculty have examined it either in general terms or in relation to health (Koetz et al., 2013; Garcia et al., 2008), most often focusing on emotional and physical strain (Dias et al., 2018; Palage et al., 2020; Costa et al., 2013) or health-related risk factors in this professional group (De Oliveira et al., 2012).

Given that both physical and psychological dimensions are main contributors to perceived QoL, factors related to spirituality and religiousness (S/R) are relevant for understanding self-reported QoL across populations, including both patients and healthy individuals (Koenig, 2012; Haraldstad et al., 2019; Borges et al., 2021). The relationship between S/R and health has been widely explored, emphasizing the interdependence of physical and mental well-being (Demir, 2019; Felicilda-Reynaldo et al., 2019; Anye et al., 2013; Goncąlves et al., 2015; Bonelli and Koenig, 2013). Elements such as faith, positive attitude, and inner peace have been associated with reduced stress and depression and are recognized as components of effective coping strategies (Panzini and Bandeira, 2007; Gardner et al., 2014).

QoL is a multidimensional construct influenced by diverse factors related to health and living conditions, and it can be strongly affected by the environmental demands faced by university professors (Dias et al., 2018; Koetz et al., 2013; de Oliveira et al., 2012; Garcia et al., 2008; Fontana and Pinheiro, 2010). From this perspective, the manifestation of S/R may influence distinct domains of QoL in this population, particularly when spirituality is understood broadly, as an individual’s search for meaning, purpose, and connection, regardless of adherence to a specific religion or institution (Koenig, 2012; Panzini et al., 2011). Exploring how S/R interacts with QoL among university professors may contribute to the design of interventions and institutional strategies aimed at improving faculty well-being.

Although a considerable body of research has examined S/R and QoL in academic contexts, most studies have focused on university students (Borges et al., 2021; Lau et al., 2015). Consequently, little is known about how these variables interact among university professors, who experience distinct personal and professional challenges. Therefore, this study aimed to assess the association between levels of S/R and QoL among university professors.

Based on the existing literature and the conceptual framework adopted, this study was guided by the following hypotheses: (H^1^) higher levels of S/R would be positively associated with overall QoL among university professors; (H^2^) Specific facets of spirituality would show the strongest correlations with the psychological domain of QoL, reflecting their role in emotional resilience and well-being; (H^3^) Sociodemographic and professional variables, such as job satisfaction, professional recognition, academic degree, and income, would contribute to QoL outcomes, but S/R would remain an independent and significant predictor even after statistical adjustment.

## 2 Methods

This cross-sectional study was conducted between February and March 2020 in accordance with the STROBE guidelines for reporting observational studies (Von Elm et al., 2008). Participants were professors from a public university in Goiás (Central-West Region), Brazil. The study protocol was approved by the Research Ethics Committee of the Medical School of the Federal University of Goiás (CAAE 66231614.1.0000.5083). All participants provided written informed consent.

### 2.1 Participants

A total of 213 university professors from a public university in Goiás State, Brazil, participated in this study. The target population comprised all 1,319 professors formally employed across the university’s four campuses and 26 academic programs, which include the exact sciences, humanities, health sciences, biological sciences, engineering, and technology.

Recruitment was carried out via institutional email invitations and departmental announcements, followed by voluntary participation. The final sample size (*n* = 213) represented approximately 16% of the total eligible faculty population, with proportional representation across all campuses and academic disciplines. All participants received detailed information about the study objectives and procedures and provided written informed consent prior to inclusion.

Eligibility criteria were: (i) being officially employed as a professor at one of the university’s campuses; (ii) being aged 18 years or older; and (iii) actively teaching at least one undergraduate or graduate course during the data collection period. Professors on academic leave, medical leave, or participating in international exchange programs at the time of recruitment were excluded.

### 2.2 Instruments and outcome variables

Data were collected through self-administered questionnaires completed in approximately 15–20 min.

#### 2.2.1 Sociodemographic data

Developed by the research team, this section included 14 questions covering personal information and professional profile. Most items were multiple-choice, addressing variables such as sex, employment type, academic degree, and individual or collective spirituality. Job satisfaction was assessed using an intensity scale ranging from “not at all satisfied” to “very satisfied.

#### 2.2.2 Spirituality and Religiousness (S/R)

S/R was measured using the WHOQOL-SRPB (Spirituality, Religiousness, and Personal Beliefs) instrument, validated for Brazil (Panzini et al., 2011). This questionnaire contains 32 items grouped into eight facets: connection to a spiritual being or force, meaning in life, awe, wholeness and integration, spiritual strength, inner peace, hope and optimism, and faith. Items are rated on a 5-point Likert scale, with higher scores indicating greater S/R. Cronbach’s alpha for the current sample was 0.960.

#### 2.2.3 Quality of Life (QoL)

QoL was assessed using the WHOQOL-BREF, also validated for Brazil (Fleck et al., 2000). This 26-item questionnaire measures four domains, Physical, Psychological, Social Relationships, and Environment, with scores ranging from 0 to 100. Higher scores indicate better QoL. Cronbach’s alpha for this study was 0.898.

### 2.3 Sample size and statistical power

An *a priori* power analysis was conducted using G*Power (Exact → Correlation: Bivariate normal model; exact distribution), assuming a two-tailed test, an expected correlation under the alternative hypothesis (*ρ* = 0.20), a significance level of *α* = 0.05, statistical power (1 − *β*) of 0.80. The analysis indicated a required total sample size of 193, with lower and upper critical r values of −0.1413 and 0.1413, respectively, and an actual power of 0.80. The final sample included 213 participants, exceeding the minimum required and thereby ensuring a power ≥ 80% to detect small correlations (approximately r = 0.20) between the variables of interest (facets of the WHOQOL-SRPB and domains of the WHOQOL-BREF).

### 2.4 Procedures

After initial invitations, data collection sessions were scheduled at times and locations convenient for participants, such as private office rooms free from distractions. Questionnaires were self-administered after researchers provided a standardized explanation of the procedure.

### 2.5 Bias and potential confounding factors

The study used a voluntary recruitment process through institutional communication channels, ensuring anonymity and confidentiality to reduce social desirability bias and response bias. Data collection took place in private and distraction-free environments, and participants completed the questionnaires independently to prevent interviewer influence.

To mitigate selection bias, proportional representation across campuses and academic areas was ensured, and inclusion/exclusion criteria were clearly defined (e.g., only active professors teaching at least one course). Additionally, measurement bias was minimized through the use of validated instruments (WHOQOL-SRPB and WHOQOL-BREF), both adapted for the Brazilian population and with excellent internal consistency (Cronbach’s *α* = 0.960 and 0.898, respectively).

Potential confounding factors, such as sex, academic degree, income, job satisfaction, and employment type, were examined using multivariate analyses (Spearman’s correlation and multiple linear regression with the stepwise method), allowing the estimation of independent associations between S/R facets and QoL domains.

### 2.6 Statistical analysis

Data normality was assessed using the Kolmogorov–Smirnov test. Associations between quality of life (QoL) domains and exploratory variables were analyzed using non-parametric tests—(Mann–Whitney U or Kruskal–Wallis), followed, when appropriate, by Nemenyi *post hoc* comparisons.

For multiple linear regression analyses, preliminary diagnostic procedures were performed to verify the assumptions of linearity, normality of residuals, and independence of errors. These were examined through residual plots, histograms, and residual sequence plots (Supplementary material). Multicollinearity was assessed using Variance Inflation Factor (VIF) and Tolerance values, adopting thresholds of VIF < 5 and Tolerance > 0.2 as acceptable.

Regression models were developed to identify predictors of WHOQOL-SRPB and WHOQOL-BREF domain scores. Variables were initially grouped into conceptual blocks (sociodemographic, occupational, and spiritual) based on theoretical rationale. Subsequently, a stepwise selection method was applied to refine the models by identifying the most statistically robust and parsimonious set of predictors. This combined approach allowed for theoretical coherence while optimizing model fit in an exploratory context.

For each model, standardized beta coefficients (*β*), 95% confidence intervals (CI), adjusted R^2^ values, and *F*-statistics (*F*(df)) were reported. All statistical analyses were conducted using IBM SPSS Statistics for Windows, Version 23.0 (IBM Corp., Armonk, NY, USA).

## 3 Results

A total of 213 university professors participated in the study. Table 1 summarizes sociodemographic characteristics and descriptive statistics for QoL and S/R measures.

**Table 1:** Sociodemographic profile of professors (*n* = 213) regarding personal and professional data, in frequency (*n*)/absolute value (%).

Variables	*n*	%	Variables	Mean	SD
Age group			WHOQOL-*brief*		
24 to 59 years	198	92.9	Physical	68.26	14.40
60 to 71 years	15	7.0	Psychological	68.99	13.79
Sex			Social relationships	66.39	15.95
Male	91	42.7	Environment	65.70	11.89
Female	122	57.3	WHOQOL-SRPB		
Area			Spiritual connection	78.76	17.65
Exactas	62	29.1	Meaning in life	84.55	12.19
Health sciences	67	31.5	Wholeness and integration	79.62	13.06
Humanities	84	39.4	Spiritual strength	76.27	14.02
Academic degree			Inner peace	80.09	14.85
Specialist	67	31.5	Hope and optimism	73.05	33.80
Master	82	38.5	Faith	77.32	13.13
Doctor	64	30.0	Awe	78.64	17.48

Mean and standard deviation (SD) for quality-of-life domains (WHOQOL-Brief) and facets of spirituality (WHOQOL-SRPB).

Table 2 presents Spearman’s correlations between WHOQOL-SRPB facets and the four WHOQOL-BREF domains. Most S/R facets showed significant positive correlations (*p* ≤ 0.01) with QoL domains. The strongest associations were observed in the psychological domain for the facets Hope and Optimism (r = 0.67), Spiritual Strength (r = 0.62), and Faith (r = 0.59), as well as for the overall SRPB score (r = 0.56).

**Table 2:** Result of the Spearman correlation between the facets of S/R from WHOQOL-SRPB and the domains of QL from WHOQOL-Brief.

	Domains WHOQOL-*bref*				
	Physical	Psychological	Social relationships	Environment	QoL overall
Facets WHOQOL-SRPB					
Spiritual connection	*r* = 0.08	*r* = 0.25	*r* = 0.19	*r* = 0.13	*r* = 0.20^*^
Meaning in life	*r* = 0.22^*^	*r* = 0.39^*^	*r* = 0.29^*^	*r* = 0.22^*^	*r* = 0.36^*^
Wholeness and integration	*r* = 0.14^*^	*r* = 0.35^*^	*r* = 0.25^*^	*r* = 0.24^*^	*r* = 0.29^*^
Spiritual strength	*r* = 0.39^*^	*r* = 0.62 ^*^^+^	*r* = 0.37^*^	*r* = 0.45^*^	*r* = 0.56 ^*^^+^
Inner peace	*r* = 0.21^*^	*r* = 0.39^*^	*r* = 0.29^*^	*r* = 0.24^*^	*r* = 0.34^*^
Hope and optimism	*r* = 0.42^*^	*r* = 0.67 ^*^^+^	*r* = 0.40^*^	*r* = 0.44^*^	*r* = 0.59 ^*^^+^
Faith	*r* = 0.32^*^	*r* = 0.59 ^*^^+^	*r* = 0.34^*^	*r* = 0.41^*^	*r* = 0.50^*^
Awe	*r* = 0.14^*^	*r* = 0.31^*^	*r* = 0.20^*^	*r* = 0.22^*^	*r* = 0.26^*^
SRPB-Overall	*r* = 0.31^*^	*r* = 0.56 ^*^^+^	*r* = 0.36^*^	*r* = 0.36^*^	*r* = 0.49^*^

^*^Significant correlations (*p* ≤ 0.01).

+ Correlations greater than 0.5.

Table 3 presents the mean (±SD) scores for the QoL domains according to sociodemographic characteristics. The Psychological and Environment domains were the most affected by these variables. Academic degree, income, and employment status were each associated with at least two QoL domains.

**Table 3:** Mean scores for WHOQOL-brief domains according to sociodemographic data.

Variables	Domains				QoL overall
	Physical	Psychological	Social relationships	Environment	
Age group^**^	*p* = 0.34	*p* < 0.001	*p* = 0.85	*p* = 0.08	*p* = 0.15
24 a 59 years	69.42 ± 14.33	71.47 ± 13.45	67.34 ± 14.55	65.70 ± 12.56	68.48 ± 11.11
60 a 71 years	69.33 ± 17.85	74.44 ± 12.59	66.11 ± 15.58	73.13 ± 12.15	70.75 ± 10.45
Academic degree^**^	*p* = 0.16	*p* = 10.03	*p* = 0.18	*p* = 0.02	*p* = 0.09
Specialist	65.94 ± 12.67	67.97 ± 11.68	65.57 ± 15.97	64.41 ± 10.36	66.28 ± 10.59
Master	69.47 ± 14.45	67.28 ± 13.61	63.82 ± 15.45	63.80 ± 10.44	66.09 ± 10.80
Doctor	69.15 ± 15.90	72.27 ± 15.59^†^	69.27 ± 13.92	69.48 ± 14.23^†^	70.04 ± 12.40
Salary income (R$)^**^	*p* = 0.02	*p* < 0.001	*p* = 0.10	*p* = 0.02	*p* = 0.01
Up to 3,000	67.40 ± 14.08	67.31 ± 13.13	62.39 ± 14.54	64.49 ± 6.81	65.40 ± 9.95
5,000 to 7,000	63.04 ± 13.83	64.05 ± 11.93	63.18 ± 19.52	61.12 ± 12.60	62.85 ± 11.20
> 7,000 R$	70.73 ± 15.93^†^	72.07 ± 15.44^†^	67.39 ± 14.92	68.79 ± 13.55^†^	69.74 ± 12.55^†^
Other appointments^*^	*p* = 0.02	*p* < 0.001	*p* = 0.18	*p* < 0.001	*p*< 0.001
No	69.61 ± 14.77	70.37 ± 14.30	66.99 ± 16.33	66.87 ± 12.37	68.46 ± 11.87
Yes	64.82 ± 12.90	65.49 ± 11.81	64.86 ± 14.96	62.71 ± 10.08	64.47 ± 9.31
Satisfaction^**^	*p* < 0.001	*p* < 0.001	*p* < 0.001	*p* < 0.001	*p* < 0.001
Very dissatisfaction	70.83 ± 15.67	67.36 ± 19.04	67.36 ± 18.96	70.57 ± 16.07	69.03 ± 15.54
Slightly dissatisfaction	62.47 ± 13.66	63.76 ± 12.91	60.53 ± 15.94	61.32 ± 10.67	62.02 ± 10.48
Satisfied	68.40 ± 15.05	68.42 ± 13.72	65.58 ± 15.74	64.52 ± 11.77	66.73 ± 10.82
Very satisfied	74.65 ± 10.03	77.74 ± 8.72^†^	75.81 ± 11.15	73.01 ± 8.52^†^	75.30 ± 7.51^†^
Work recognition^**^	*p* < 0.001	*p* < 0.001	*p* < 0.001	*p* < 0.001	*p* < 0.001
None	61.31 ± 21.36	60.42 ± 21.69	65.57 ± 15.97	63.54 ± 14.88	60.21 ± 17.81
Little	61.79 ± 14.11	62.08 ± 15.56	57.08 ± 17.96	60.78 ± 11.12	60.43 ± 11.59
Much	72.51 ± 14.37	75.21 ± 11.39	75.42 ± 13.41^†^	71.19 ± 10.90	73.58 ± 9.71
Extremely	83.04 ± 3.42^†^	83.33 ± 9.62^†^	70.83 ± 8.33	78.91 ± 16.21^†^	79.03 ± 8.57^†^
Individual S/R^**^	*p* = 0.06	*p* < 0.001	*p* < 0.002	*p* = 0.46	*p* = 0.01
No	61.37 ± 16.08	65.03 ± 14.58	55.95 ± 19.75	64.62 ± 14.18	61.74 ± 12.04
1 to 3 times/week	66.46 ± 16.14	64.67 ± 12.04	68.12 ± 15.00	62.50 ± 7.30	65.44 ± 8.50
Everyday	70.05 ± 13.21^†^	72.72 ± 12.63^†^	69.26 ± 14.92^†^	66.83 ± 11.20	69.71 ± 10.94^†^

^*^Mann–Whitney; ^**^Kruskal-Wallis.

† Nemenyi.

Application of multiple linear regression (Table 4) to assess the influence of E/R facets on overall QoL (WHOQOL-BREF) showed that “Hope and Optimism” and “Spiritual Strength” were significant. The model, including these facets, explained 28% of the variance in overall QoL (adjusted *R*^2^ = 0.28).

**Table 4:** Multiple linear regression (stepwise method) between the facets of WHOQOL-SRPB and overall QoL from WHOQOL-Brief, highlighting the adjusted *R*^2^ and confidence interval (CI 95%) for each variable and the total overall influence of the facets.

Predictors	Adjusted *R*^2^	*β*	SE	*t*	*p*	CI 95%
Spiritual connection	0.04	0.06	0.07	0.88	0.38	(−0.08–0.21)
Meaning in life	0.11	0.01	0.07	0.12	0.9	(−0.13–0.15)
Wholeness and integration	0.04	0.05	0.07	0.77	0.44	(−0.09–0.19)
Spiritual strength	0.21	0.25	0.1	2.6	0.01	(0.05–0.45)
Inner peace	0.09	0.01	0.09	0.06	0.95	(−0.17–0.19)
Hope and optimism	0.25	0.26	0.08	3.29	<0.001	(0.11–0.42)
Faith	0.21	0.13	0.08	1.6	0.11	(−0.03–0.29)
Awe	0.05	0.01	0.07	0.18	0.86	(−0.13–0.15)
Overall QoL	0.28					

The multiple linear regression analysis (stepwise) identified significant predictors of QoL among university professors (Table 5). Professional recognition was the most consistent factor, positively influencing all domains and overall QoL (*β* ranging from 5.69 to 7.96, *p* < 0.01), followed by job satisfaction, which was also relevant across four domains (*p* < 0.05). Subjective and demographic factors proved to be crucial: personal belief had the most pronounced contribution in the Social Relationships domain (*β* = 27.94, *p* = 0.002), while professors aged 41 or older reported greater psychological well-being (*β* = 7.39, *p* < 0.001). The explanatory power of the models (adjusted *R*^2^ from 0.06 to 0.19) indicates that while these predictors are central, other elements also influence the quality of life in this group.

**Table 5:** Multiple linear regression (stepwise method) between the sociodemographic profile and results of WHOQOL-Brief (*n* = 213).

Predictors	Adjusted *R*^2^	*β*	SE	*t*	*p*	CI 95%
Physical domain						
Professional recognition	0.11	5.85	2.39	2.44	0.015	(1.13–10.57)
Sex (Male)		4.77	1.90	2.51	0.013	(1.02–8.52)
Individual spirituality		7.15	2.86	2.50	0.013	(1.52–12.79)
Job satisfaction		4.65	2.09	2.23	0.027	(0.53–8.76)
Psychological domain						
Age (41 or mor)	0.16	7.39	1.75	4.21	0.000	(3.93–10.84)
Professional recognition		7.37	2.16	3.41	0.001	(3.11–11.63)
Job satisfaction		4.52	1.93	2.34	0.020	(0.71–8.34)
Social relationships domain						
Professional recognition	0.19	7.96	2.58	3.08	0.002	(2.86–13.05)
Personal belief		27.94	8.97	3.11	0.002	(10.25–45.62)
Job satisfaction		5.58	2.21	2.52	0.012	(1.21–9.94)
Individual spirituality		7.30	3.11	2.34	0.020	(1.16–13.43)
Exact Sciences		−4.73	2.18	2.17	0.031	(−9.02 - -0.43)
Environment domain						
Professional recognition	0.06	5.69	1.93	2.94	0.004	(1.88–9.49)
Other activities		−4.07	1.77	−2.30	0.023	(−7.57 - -0.58)
Exact Sciences		−3.53	1.75	−2.02	0.045	(−6.99 - -0.08)
QoL overall						
Professional recognition	0.16	6.54	1.83	3.57	0.000	(2.93–10.15)
Age (41 or mor)		3.21	1.45	2.21	0.028	(0.35–6.08)
Job satisfaction		4.52	1.60	2.82	0.005	(1.36–7.68)
Personal belief		14.06	6.31	2.23	0.027	(1.61–26.5)

CI, confidence interval; SE, standard deviation.

The multiple linear regression analysis using the stepwise method (Table 6) identified high-influence predictors for facets of S/R, with models exhibiting substantial explanatory power (adjusted *R*^2^ up to 0.37). Religious practice and individual spirituality were the most robust predictors; individual spirituality for Faith (*β* = 0.85, *p* < 0.001) and service attendance for Spiritual Connection (*β* = 0.73, *p* < 0.001). Notably, leadership recognition was a consistent, positive predictor for Spiritual Connection (*β* = 0.55, *p* < 0.001), Meaning in Life (*β* = 0.25, *p* = 0.012), and Faith (*β* = 0.46, *p* < 0.001). Factors such as age (41 + years) and years of teaching were also associated with greater Inner Peace (*β* = 0.36, *p* < 0.001) and Hope (*β* = 0.28, *p* = 0.001), respectively. In contrast, the ‘Admiration’ facet exhibited low explanatory power (R^2^ = 0.02).

**Table 6:** Multiple linear regression (stepwise method) between the sociodemographic profile and results of WHOQOL-SRPB (*n* = 213).

Predictor	Adjusted *R*^2^	*β*	SE	*t*	*p*	CI 95%
Spiritual connection						
Individual spirituality	0.37	0.80	0.15	5.32	0.000	(0.51–1.09)
Attends religious services		0.73	0.13	5.76	0.000	(0.48–0.98)
Leadership recognition		0.55	0.13	4.26	0.000	(0.31–0.81)
Personal belief		1.04	0.45	2.30	0.022	(0.15–1.94)
Meaning in life						
Attends religious services	0.16	0.48	0.10	4.85	0.000	(0.28–0.67)
Leadership recognition		0.25	0.10	2.54	0.012	(0.06–0.45)
Health sciences		0.20	0.08	2.37	0.019	(0.03–0.36)
Has children		0.16	0.08	2.03	0.044	(0.01–0.32)
Wholeness and integration						
Attends religious services	0.26	0.57	0.11	5.34	0.000	(0.36–0.78)
Leadership recognition		0.44	0.11	4.00	0.000	(0.22–0.65)
Age (41 or mor)		0.28	0.08	3.35	0.001	(0.12–0.45)
Individual spirituality		0.32	0.12	2.53	0.012	(0.07–0.56)
Job satisfaction		0.18	0.09	2.04	0.043	(0.01–0.36)
Spiritual strength						
Personal belief	0.33	1.56	0.39	3.98	0.000	(0.79–2.34)
Attends religious services		0.62	0.11	5.61	0.000	(0.41–0.83)
Individual spirituality		0.40	0.13	3.08	0.002	(0.14–0.65)
Leadership recognition		0.34	0.11	3.05	0.003	(0.12–0.57)
Inner peace						
Professional recognition	0.17	0.42	0.11	3.78	0.000	(0.21–0.64)
Age (41 or mor)		0.36	0.09	3.96	0.000	(0.18–0.55)
Attends religious services		0.44	0.12	3.78	0.000	(0.21–0.67)
Hope and optimism						
Time in teaching_11 or more	0.12	0.28	0.08	3.33	0.001	(0.12–0.45)
Attends religious services		0.36	0.11	3.30	0.001	(0.14–0.57)
Professional recognition		0.27	0.10	2.66	0.008	(0.07–0.48)
Faith						
Individual spirituality	0.36	0.85	0.15	5.68	0.000	(0.55–1.14)
Attends religious services		0.61	0.13	4.76	0.000	(0.35–0.86)
Leadership recognition		0.46	0.13	3.55	0.000	(0.21–0.72)
Personal belief		1.34	0.45	2.96	0.003	(0.45–2.24)
Awe						
Professional recognition	0.02	0.25	0.11	2.34	0.015	(0.04–0.47)
Health sciences		0.17	0.08	2.37	0.019	(0.03–0.36)
Has children		0.44	0.10	2.04	0.044	(0.01–0.32)

CI, confidence interval; SE, standard deviation.

## 4 Discussion

Based on the QoL data measured using the WHOQOL-BREF instrument, higher levels of S/R, as assessed by the WHOQOL-SRPB, were associated with more positive perceptions of QoL among the professors in this study. The significant positive correlations between WHOQOL-SRPB facets and WHOQOL-BREF domains confirm that these variables tend to vary linearly in the same direction. The strongest correlations were observed in the psychological domain of QoL. Beyond the bivariate analyses, the coefficients of determination and multiple linear regression results demonstrated a positive influence of S/R facets on QoL scores, with hope/optimism, spiritual strength, and faith emerging as the most influential predictors.

These positive associations between S/R and QoL outcomes appear particularly relevant for overall well-being, especially within the psychological domain, as university professors are vulnerable to mental health disorders such as depression, anxiety, stress, panic, and burnout (Koetz et al., 2013; Costa et al., 2013; Fontana and Pinheiro, 2010). This finding aligns with previous literature positioning S/R components as key pillars of resilience, suggesting that spiritual belief systems provide a framework of meaning and purpose that enables individuals to cognitively reappraise adverse events (Koenig, 2009). Within the academic context, this framework may empower professors to reinterpret daily challenges as opportunities for personal growth and social contribution, thereby mitigating the psychological manifestations of occupational stress.

There is a clear overlap between psychological QoL and mental disorders such as stress and depression, both of which tend to be lower among individuals with higher levels of S/R. Several studies with university students have confirmed this association, reporting that greater S/R are linked to higher psychological and overall QoL (Bonelli and Koenig, 2013; Chai et al., 2012; Pillay et al., 2016; Casu et al., 2018). Similar evidence has been observed in mixed samples, including adults and older adults, whether healthy or ill, where higher levels of S/R were associated with better psychological QoL and lower stress and depression, with facets such as peace, meaning, optimism, and happiness playing crucial roles (Peres et al., 2017; Vitorino et al., 2018). In our study, most S/R facets, as well as the total WHOQOL-SRPB score, were positively correlated with WHOQOL-BREF domains, particularly the Psychological, Social Relationships, and Environment domains, as well as overall QoL. The strongest associations were again found for spiritual strength, hope/optimism, and faith.

The multiple linear regression analysis confirmed these results, indicating that spiritual strength, hope/optimism, and faith were the strongest predictors of overall QoL, explaining 28% (*R*^2^ = 0.28) of its variance. This magnitude is consistent with findings from a meta-analysis by Sawatzky, Ratner, and Chiu (Sawatzky et al., 2005), which reported an *R*^2^ = 0.27 across 51 studies evaluating the relationship between spirituality and QoL in diverse populations and health conditions.

Sociodemographic and professional variables also played an important role in QoL outcomes. According to the WHOQOL-BREF results, professors with doctoral degrees reported significantly higher scores (*p* < 0.05) in the Psychological and Environment domains compared to those with master’s or specialist qualifications. This pattern mirrored the effect of income: participants with higher salaries scored significantly higher (*p* < 0.05) in three of the four QoL domains (Physical, Psychological, and Environment) and in overall QoL. These findings are consistent with the results reported by Koetz, Rempel, and Perico (Koetz et al., 2013) among 203 university faculty members in southern Brazil.

Similarly, Dias et al. (2018) found that professors who were more satisfied with their income, teaching activities, autonomy, and professional standing reported higher QoL at work. Thus, among university faculty, individual achievements, academic status, and perceptions of professional recognition appear to be important influence on QoL outcomes. These factors may contribute to lower scores in the Environment and Social Relationships domains among professors with lower income or academic qualifications.

Professors holding multiple employment contracts exhibited lower QoL scores than those working exclusively at the studied university. Previous studies have reported similar patterns, showing that long working hours and multiple jobs are linked to poorer physical and psychological QoL among university faculty (De Oliveira et al., 2012; Fontana and Pinheiro, 2010; Gomes et al., 2017; Martinez et al., 2009). The diversity of employment arrangements in higher education, including temporary, permanent, and part-time contracts, creates varying levels of income, job stability, and career prospects, which may in turn influence feelings of security, opportunity, and self-esteem. Given that higher individual S/R levels were associated with better QoL, these findings suggest that personal beliefs could potentially serve as a protective factor in such contexts.

Some other sociodemographic variables had a positive contribution on WHOQOL-brief scores, such as older age, job satisfaction, professional recognition, and higher levels of individual S/R, particularly in the Psychological and Environment domains. Evidence in the literature supports the positive correlation between age and QoL among university professors (Sanchez et al., 2019). Likewise, studies have highlighted the influence of feelings of satisfaction, professional achievement, and recognition among peers in this context (Dias et al., 2018; Koetz et al., 2013). For younger professors, periods of uncertainty and adaptation during the early years in the workforce often coincide with other transitional aspects of personal life. Different stages of job stability, personal expectations, personal maturity, lifestyle, and other multiple factors are likely to influence these outcomes (Dias et al., 2018; Koetz et al., 2013).

Thus, among university professors, several combined factors, including academic degree, income, age, professional roles, leadership recognition, and employment type, appear to influence the observed quality of life outcomes. These factors are themselves affected by both personal and professional aspects, such as sense of security, financial resources, access to healthcare opportunities, self-esteem, positive feelings, higher levels of spirituality and religiosity, among others assessed through the items of the WHOQOL-bref and WHOQOL-SRPB instruments (Panzini et al., 2011; Fleck et al., 2000).

These findings are in line with those reported by Palage et al. (2020), in which variables such as employment contract, salary income, academic degree, among others, were associated with symptoms of burnout syndrome (overall mean of 33.5%) in 113 professors at a state university in Minas Gerais, Brazil, as indicated by high scores in emotional exhaustion and depersonalization (social relationship difficulties) linked to low levels of personal accomplishment. Aspects of personal and professional achievement among teachers determine psychological components that predict well-being and QoL, as observed in the study by Damásio, Melo and Silva (Damásio et al., 2013) with 517 primary education teachers. Having positive feelings toward personal and professional life, along with internal motivation to cope with external challenges, were identified as key conditions for the QoL construct, and these factors might be associated with stronger personal beliefs connected to higher S/R.

Importantly, much of the positive influence of S/R on QoL does not necessarily depend on religious affiliation, as suggested by prior reviews (Vitorino et al., 2018; Counted et al., 2018). Several studies involving university students have shown that spirituality, rather than religious affiliation, is the most decisive variable for better QoL outcomes (Lau et al., 2015). The WHOQOL-SRPB instrument used in this study focuses on personal beliefs rather than institutional religiosity, allowing for a broader assessment of spiritual dimensions. Professors who reported engaging in individual spiritual practices demonstrated higher scores in the Psychological, Social Relationships, and overall QoL domains compared to those without such practices (Table 3). Furthermore, higher levels of individual S/R showed a strong correlation with improved scores on the WHOQOL-SRPB instrument (Table 6).

As seen in other populations, whether healthy or with chronic conditions, spirituality and personal beliefs may serve as valuable protective and coping mechanisms against the occupational pressures that commonly affect university professors (Koenig, 2012; Vitorino et al., 2018; Damásio et al., 2013; De Gonçalves et al., 2017).

This study makes a significant contribution by identifying a positive association between S/R and quality of life among university professors, an aspect that has not been widely explored in the existing literature. While previous research has focused on patient populations, here we present favorable outcomes for adults who are actively engaged in their professional roles, highlighting spirituality as a factor that may contribute meaningfully to psychological well-being, social relationships, and environmental perception among these educators. Moreover, our analysis considered how these professors manage their social interactions, suggesting that spirituality may play a crucial role in relationships with colleagues and students, and reinforcing the importance of strategies such as hope, optimism, and faith for coping with the challenges of academic life and enhancing resilience when necessary.

### 4.1 Limitations

Its cross-sectional and observational design limits interpretation to associations between variables, rather than cause-and-effect relationships. Additionally, the use of self-reported questionnaires inherently introduces a degree of subjectivity. Variations in religious and spiritual adherence could also influence perceptions of S/R and QoL. Additionally, although the sample was drawn from a single professional group, internal heterogeneity exists across variables such as employment status (temporary vs. permanent), exclusivity of university employment, academic qualifications, and income levels. Another aspect is that, since the study was conducted at a state public university, the results may not be generalizable to professors working at private or federal institutions.

Social desirability bias may have distorted some responses, compromising the validity of the data. Furthermore, the invariance of the WHOQOL-SRPB instrument should be assessed with caution, as variations in interpretation may shape comparisons between groups. Conducting multiple statistical tests increases the likelihood of significant results occurring by chance, and we are aware that corrections may be required in future replications of this type of research. Data collection, carried out between January and February 2020, took place just before the COVID-19 pandemic, which may differ considerably from the post-pandemic context.

## 5 Conclusion

Positive associations were identified between S/R and QoL among university professors. Intrinsic spirituality and personal beliefs, particularly those reflected in the facets hope/optimism, spiritual strength, and faith, emerged as key factors associated with higher QoL. The strongest relationships were observed in the Psychological, Environment, and Social Relationships domains, which were also influenced by sociodemographic and professional variables such as academic degree, income, job satisfaction, and employment contract type.

These findings suggest that fostering spiritual and meaning-centered dimensions may serve as a protective factor against common mental health challenges faced by university professors. Interventions based on spiritual and psychological dimensions, such as secular mindfulness programs, development of psychological capital (hope, resilience, optimism), and reflective discussion groups on purpose and vocation, may offer promising strategies to promote mental health and enhance QoL in this professional group. Further observational and interventional studies are warranted to strengthen the evidence on the positive relationship between S/R and health-related QoL among university faculty.

## Data Availability

The raw data supporting the conclusions of this article will be made available by the authors, without undue reservation.
