# Supplementary material for: Quality of life in university professors: association with components of spirituality and professional achievement
Source: Front Psychol. 2026 Jan 5;16:1735377. doi: 10.3389/fpsyg.2025.1735377 (PMC12813167; doi:10.3389/fpsyg.2025.1735377)
Supplement: Supplementary file 1 [file Data_Sheet_1.pdf]

SUPPLEMENTARY MATERIAL

Graphical analyses for the diagnosis of linearity, normality, and independence of residuals.

Figure 01 - Plot to assess linearity

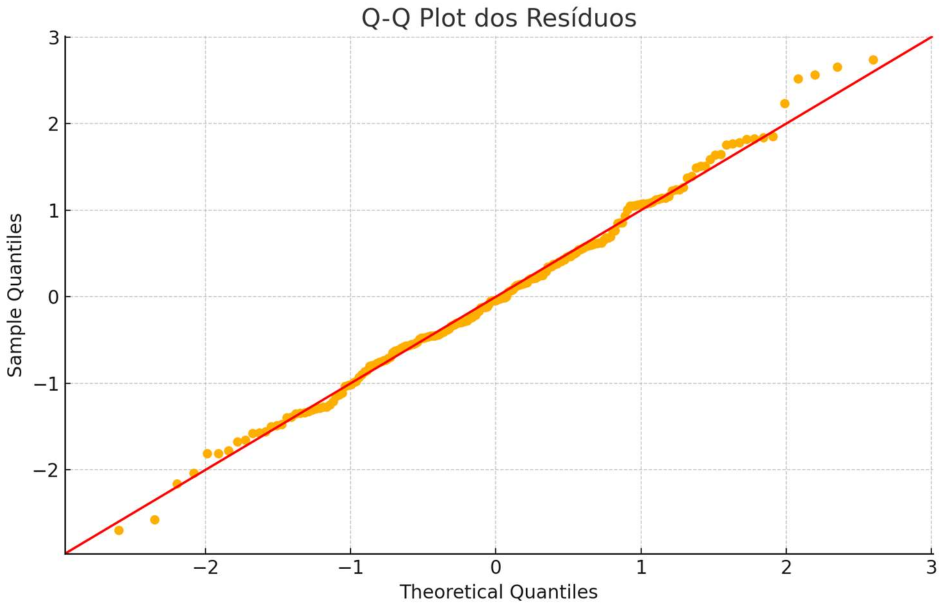

Figure 02 - Histogram and Q-Q plot of residuals (normality)

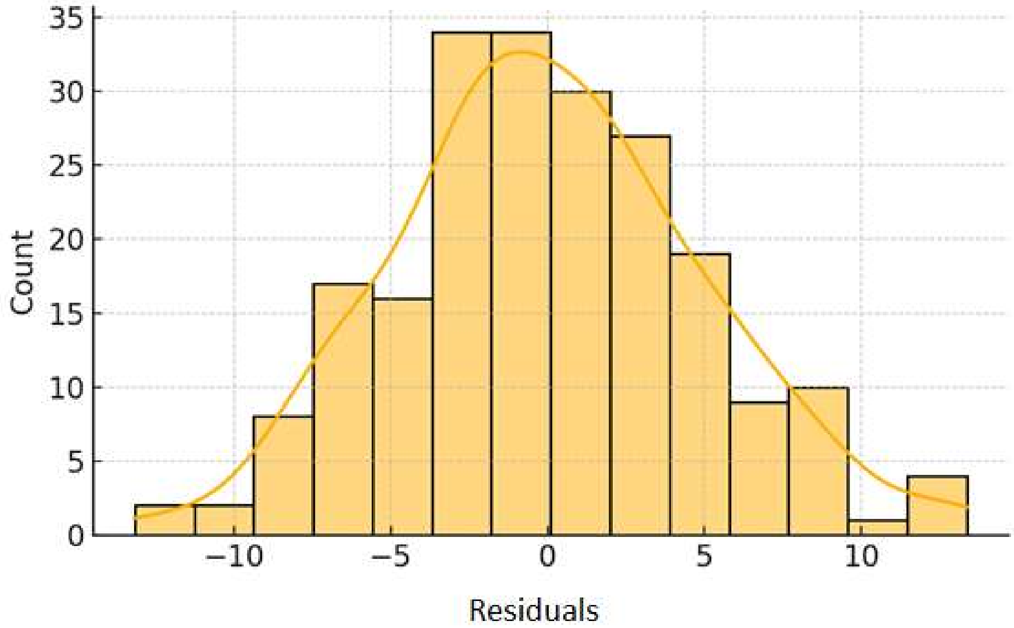

Figure 03 - Durbin-Watson Test Plot (independence from residuals)

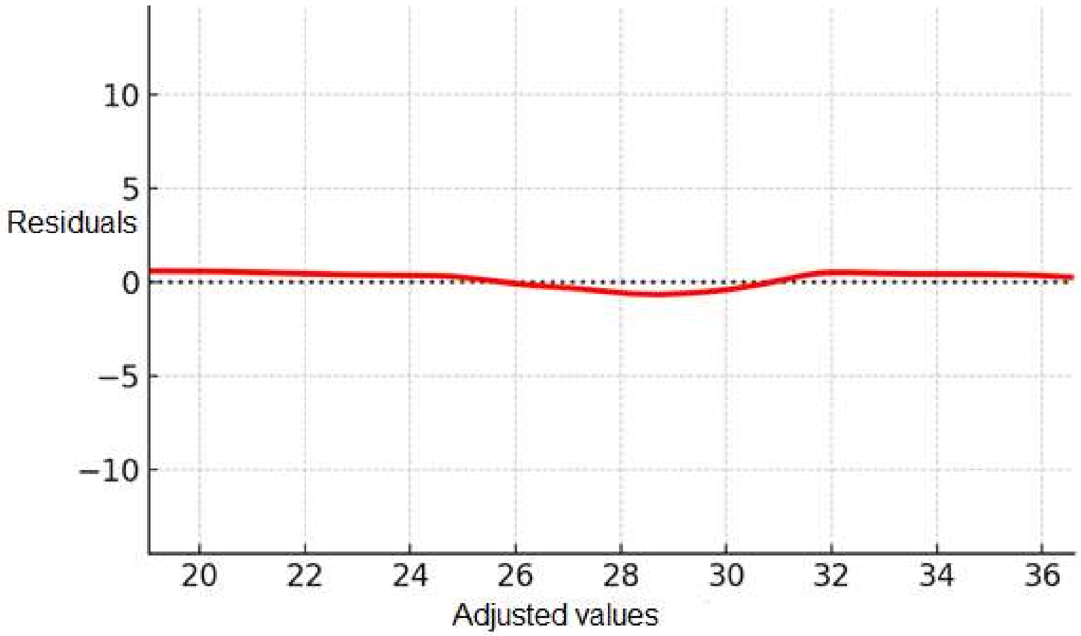

Durbin-Watson statistic:
Value = 2.11, indicating no autocorrelation of residuals (ideal: ~2)
